# (*E*)-*N*′-(4-Meth­oxy­benzyl­idene)pyridine-3-carbohydrazide dihydrate

**DOI:** 10.1107/S1600536813017406

**Published:** 2013-06-29

**Authors:** J. Josephine Novina, G. Vasuki, M. Suresh, M. Syed Ali Padusha

**Affiliations:** a Department of Physics, Idhaya College for Women, Kumbakonam-1, India; b Department of Physics, Kunthavai Naachiar Govt. Arts College (W) (Autonomous), Thanjavur-7, India; c PG & Research Department of Chemistry, Jamal Mohamed College, Tiruchirappalli-20, India

## Abstract

In the title compound, C_14_H_13_N_3_O_2_·2H_2_O, the hydrazone mol­ecule adopts an *E* conformation with respect to the C=N bond. The dihedral angle between the benzene and pyridine rings is 8.55 (10)°. The methyl­idene–hydrazide [–C(=O)–N–N=C–] fragment is essentially planar, with a maximum deviation of 0.0375 (13) Å. The mean planes of the benzene and pyridine rings make dihedral angles of 2.71 (14) and 11.25 (13)°, respectively, with mean plane of the methyl­idene-hydrazide fragment. In the crystal, the benzohydrazide and water mol­ecules are linked by N—H⋯O, O—H⋯O and O—H⋯N hydrogen bonds into a three-dimensional network.

## Related literature

For the biological activity of benzohydrazides, see: Hai-Yun (2011 ▶); Havanur *et al.* (2010 ▶); Parashar *et al.* (2009 ▶). For details of the ability of benzohydrazone compounds to inhibit cell growth and DNA synthesis, see: Ambwani *et al.* (2011 ▶); Despaigne *et al.* (2010 ▶); Havanur *et al.* (2010 ▶). For background to the use of benzohydrazides as catalysts, see: Seleem *et al.* (2011 ▶); Singh & Raghav (2011 ▶). For related structures, see: Ahmad *et al.* (2010 ▶); Hu & Liu (2012 ▶); Shi & Li (2012 ▶).
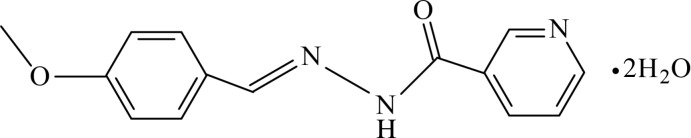


## Experimental

### 

#### Crystal data

C_14_H_13_N_3_O_2_·2H_2_O
*M* *_r_* = 291.31
Monoclinic, 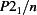

*a* = 7.6534 (6) Å
*b* = 16.3503 (11) Å
*c* = 11.4887 (6) Å
β = 96.889 (2)°
*V* = 1427.26 (17) Å^3^
*Z* = 4
Mo *K*α radiation
μ = 0.10 mm^−1^
*T* = 296 K
0.30 × 0.25 × 0.20 mm

#### Data collection

Bruker Kappa APEXII CCD diffractometer
Absorption correction: multi-scan (*SADABS*; Bruker, 2004 ▶) *T*_min_ = 0.970, *T*_max_ = 0.980
11391 measured reflections
3449 independent reflections
2050 reflections with *I* > 2σ(*I*)
*R*_int_ = 0.028

#### Refinement

*R*[*F* ^2^ > 2σ(*F* ^2^)] = 0.046
*wR*(*F* ^2^) = 0.155
*S* = 0.93
3449 reflections
206 parameters
6 restraints
H atoms treated by a mixture of independent and constrained refinement
Δρ_max_ = 0.21 e Å^−3^
Δρ_min_ = −0.17 e Å^−3^

### 

Data collection: *APEX2* (Bruker, 2004 ▶); cell refinement: *APEX2* and *SAINT* (Bruker, 2004 ▶); data reduction: *SAINT* and *XPREP* (Bruker, 2004 ▶); program(s) used to solve structure: *SIR92* (Altomare *et al.*, 1994 ▶); program(s) used to refine structure: *SHELXL97* (Sheldrick, 2008 ▶); molecular graphics: *ORTEP-3 for Windows* (Farrugia, 2012 ▶) and *Mercury* (Macrae *et al.*, 2008 ▶); software used to prepare material for publication: *PLATON* (Spek, 2009 ▶).

## Supplementary Material

Crystal structure: contains datablock(s) I, global. DOI: 10.1107/S1600536813017406/sj5336sup1.cif

Structure factors: contains datablock(s) I. DOI: 10.1107/S1600536813017406/sj5336Isup2.hkl

Supplementary material file. DOI: 10.1107/S1600536813017406/sj5336Isup3.cml

Additional supplementary materials:  crystallographic information; 3D view; checkCIF report

## Figures and Tables

**Table 1:** Hydrogen-bond geometry (Å, °)

*D*—H⋯*A*	*D*—H	H⋯*A*	*D*⋯*A*	*D*—H⋯*A*
N2—H2*N*2⋯O1*W*	0.86	2.08	2.9013 (17)	161
O1*W*—H1*O*1⋯N3^i^	0.81 (1)	2.08 (1)	2.8697 (18)	166 (2)
O1*W*—H2*O*1⋯O2*W* ^ii^	0.83 (1)	1.93 (1)	2.749 (2)	171 (2)
O2*W*—H1*O*2⋯N1^iii^	0.83 (2)	2.40 (2)	3.209 (2)	166 (3)
O2*W*—H2*O*2⋯O2	0.83 (2)	2.01 (2)	2.8182 (17)	164 (2)

Symmetry codes: (i) 
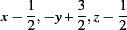
; (ii) 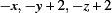; (iii) 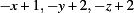.

**Fig. 1.**
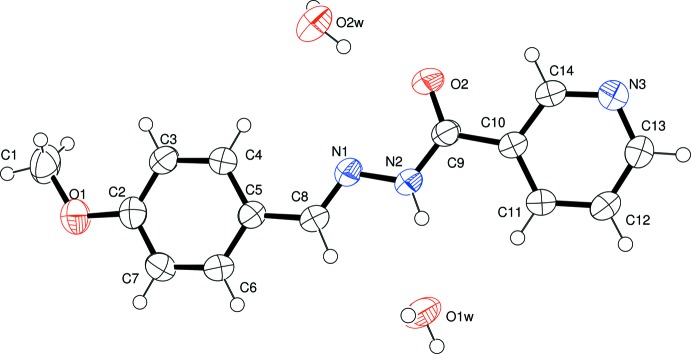
The molecular structure of the title molecule, with the atom labelling. Displacement ellipsoids are drawn at the 50% probability level.

**Fig. 2.**
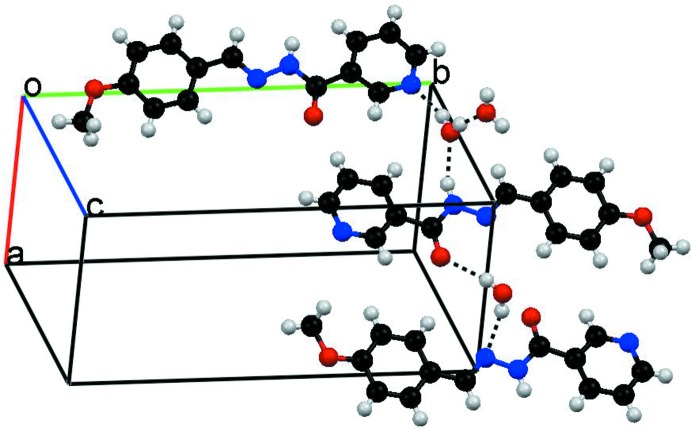
Crystal packing of the title compound viewed along the a axis. Hydrogen bonds are shown as dashed lines.

## supplementary crystallographic information

### Comment

Hydrazones have attracted much attention for their excellent biological properties, such as antimicrobial, anti-convulsant, analgestic, anti-inflammatory, antiplatelet, antitubercular, anticancer, antitumor (Hai-Yun, 2011), antiviral and vasodilator activities (Parashar *et al.*, 2009). Hydrazones possessing azomethine –NHN═CH– groups constitute an important class of compounds for new drug development (Hai-Yun, 2011). Moreover, hydrazones derived from 2-acetylpyridine are known to inhibit the proliferation of tumour cells to a greater extent compared to standard anticancer agents (Havanur *et al.*, 2010). In addition, metal complexes with hydrazones exhibit antimicrobial, DNA-binding and cytotoxic activities. It has also been shown that these metal complexes can be potent inhibitors of cell growth and DNA synthesis (Despaigne *et al.*, 2010; Havanur *et al.*, 2010; Ambwani *et al.*, 2011). Metal complexes with hydrazones also have potential applications as catalysts, luminescent probes and molecular sensors (Seleem *et al.*, 2011; Singh & Raghav, 2011). We report herein the crystal structure of the title compound, a new hydrazone.

The title compound (Fig. 1), C_14_H_13_N_3_O_2_.2H_2_O, comprises one benzohydrazide molecule and two water molecules. The hydrazone molecule adopts an E conformation with respect to the C═N bond with the torsion angle of -177.41 (16)° (C8—N1—N2—C9). Phenyl and pyridine rings (C2—C7 and N3/C10—C14, respectively) are each planner with a dihedral angle 8.55 (10)° between their mean-planes. The methylidenehydrazide fragment O2/C9/N2/N1/C8 in the title compound is essentially planar with maximum deviation being -0.0375 (13) Å for the N1 atom. The mean-planes of the benzene and pyridine rings make dihedral angles of 2.71 (14)° and 11.25 (13)°, respectively, with mean–plane of the methylidenehydrazide fragment. The C8═N1 and C9═O2 bond lengths are 1.270 (2) and 1.2199 (18) Å, respectively, which is very close to the values found in related structures (Hu & Liu, 2012; Shi & Li, 2012; Ahmad *et al.*, 2010). The methoxy group is co–planar with the benzene ring to which it is bound with the C1—O1—C2—C3 torsion angle = -0.26 (27)°.

In the crystal packing (Fig. 2), the molecules of benzohydrazide and water are linked by N2—H2N2···O1W, O1W—H2O1···O2W, O2W—H2O2···O2, O1W—H1O1···N3 and O2W—H1O2···N1 hydrogen bonds (Table 1) into a three–dimensional network.

### Experimental

Anisaldehyde (1.2 ml, 0.01 mol) and benzoic acid hydrazide (1.37 g, 0.01 mol) were added to ethanol(10 ml) of and stirred for an hour in the presence of hydrochloric acid to form a white precipitate. The precipitate was washed with sodium bicarbonate solution and filtered and again washed with petroleum ether (40–60%)and dried in air. The compound was recrystallized from absolute ethanol.

### Refinement

H atoms were placed in geometrically idealized positions and constrained to ride on their parent atoms with C—H = 0.93 Å, CH_3_ = 0.96 Å, N—H = 0.86 Å and O—H = 0.81–0.83 Å with *U*_iso_(H) = 1.5*U*_eq_(CH_3_) and 1.2*U*_eq_(CH, NH).

### Crystal data

**Table:**

C_14_H_13_N_3_O_2_·2H_2_O	*F*(000) = 616
*M**_r_* = 291.31	*D*_x_ = 1.356 Mg m^−^^3^
Monoclinic, *P*2_1_/*n*	Mo *K*α radiation, λ = 0.71073 Å
Hall symbol: -P 2yn	Cell parameters from 2772 reflections
*a* = 7.6534 (6) Å	θ = 5.0–49.6°
*b* = 16.3503 (11) Å	µ = 0.10 mm^−^^1^
*c* = 11.4887 (6) Å	*T* = 296 K
β = 96.889 (2)°	Block, colorless
*V* = 1427.26 (17) Å^3^	0.30 × 0.25 × 0.20 mm
*Z* = 4	

### Data collection

**Table:**

Bruker Kappa APEXII CCD diffractometer	3449 independent reflections
Radiation source: fine-focus sealed tube	2050 reflections with *I* > 2σ(*I*)
Graphite monochromator	*R*_int_ = 0.028
ω and φ scan	θ_max_ = 28.2°, θ_min_ = 3.0°
Absorption correction: multi-scan (*SADABS*; Bruker, 2004)	*h* = −6→10
*T*_min_ = 0.970, *T*_max_ = 0.980	*k* = −21→21
11391 measured reflections	*l* = −13→15

### Refinement

**Table:**

Refinement on *F*^2^	Secondary atom site location: difference Fourier map
Least-squares matrix: full	Hydrogen site location: inferred from neighbouring sites
*R*[*F*^2^ > 2σ(*F*^2^)] = 0.046	H atoms treated by a mixture of independent and constrained refinement
*wR*(*F*^2^) = 0.155	*w* = 1/[σ^2^(*F*_o_^2^) + (0.0961*P*)^2^] where *P* = (*F*_o_^2^ + 2*F*_c_^2^)/3
*S* = 0.93	(Δ/σ)_max_ = 0.001
3449 reflections	Δρ_max_ = 0.21 e Å^−^^3^
206 parameters	Δρ_min_ = −0.17 e Å^−^^3^
6 restraints	Extinction correction: *SHELXL97* (Sheldrick, 2008), Fc^*^=kFc[1+0.001xFc^2^λ^3^/sin(2θ)]^-1/4^
Primary atom site location: structure-invariant direct methods	Extinction coefficient: 0.009 (2)

### Special details

**Table:**

Geometry. All e.s.d.'s (except the e.s.d. in the dihedral angle between two l.s. planes) are estimated using the full covariance matrix. The cell e.s.d.'s are taken into account individually in the estimation of e.s.d.'s in distances, angles and torsion angles; correlations between e.s.d.'s in cell parameters are only used when they are defined by crystal symmetry. An approximate (isotropic) treatment of cell e.s.d.'s is used for estimating e.s.d.'s involving l.s. planes.
Refinement. Refinement of *F*^2^ against ALL reflections. The weighted *R*-factor *wR* and goodness of fit *S* are based on *F*^2^, conventional *R*-factors *R* are based on *F*, with *F* set to zero for negative *F*^2^. The threshold expression of *F*^2^ > σ(*F*^2^) is used only for calculating *R*-factors(gt) *etc*. and is not relevant to the choice of reflections for refinement. *R*-factors based on *F*^2^ are statistically about twice as large as those based on *F*, and *R*- factors based on ALL data will be even larger.

### Fractional atomic coordinates and isotropic or equivalent isotropic displacement parameters (Å^2^)

**Table:**

	*x*	*y*	*z*	*U*_iso_*/*U*_eq_	
O1W	−0.21558 (17)	0.92875 (9)	0.70457 (12)	0.0661 (4)	
O1	0.21045 (18)	1.39338 (7)	0.86316 (12)	0.0652 (4)	
O2W	0.4378 (2)	1.01711 (9)	1.14079 (13)	0.0670 (4)	
N1	0.15479 (18)	1.00258 (8)	0.89482 (11)	0.0459 (4)	
N2	0.09417 (18)	0.92297 (7)	0.87676 (11)	0.0449 (4)	
H2N2	0.0061	0.9121	0.8253	0.054*	
N3	0.1583 (2)	0.63790 (8)	0.97900 (12)	0.0530 (4)	
O2	0.29733 (18)	0.87747 (7)	1.01980 (11)	0.0688 (5)	
C1	0.3384 (3)	1.42723 (12)	0.9485 (2)	0.0717 (6)	
H1A	0.3071	1.4158	1.0253	0.108*	
H1B	0.3438	1.4853	0.9375	0.108*	
H1C	0.4512	1.4035	0.9408	0.108*	
C2	0.1867 (2)	1.31049 (10)	0.86152 (14)	0.0470 (4)	
C3	0.2804 (2)	1.25610 (10)	0.93809 (15)	0.0477 (4)	
H3	0.3671	1.2753	0.9952	0.057*	
C4	0.2445 (2)	1.17334 (10)	0.92929 (14)	0.0460 (4)	
H4	0.3080	1.1372	0.9806	0.055*	
C5	0.1152 (2)	1.14332 (9)	0.84510 (13)	0.0413 (4)	
C8	0.0684 (2)	1.05745 (10)	0.83445 (14)	0.0450 (4)	
H8	−0.0278	1.0421	0.7818	0.054*	
C9	0.1777 (2)	0.86340 (9)	0.94232 (13)	0.0422 (4)	
C10	0.1187 (2)	0.77765 (9)	0.91597 (13)	0.0384 (4)	
C14	0.1898 (2)	0.71787 (10)	0.99233 (13)	0.0467 (4)	
H14	0.2650	0.7345	1.0577	0.056*	
C13	0.0525 (2)	0.61574 (10)	0.88326 (15)	0.0520 (5)	
H13	0.0303	0.5603	0.8708	0.062*	
C6	0.0240 (2)	1.19971 (10)	0.76869 (14)	0.0482 (4)	
H6	−0.0629	1.1809	0.7114	0.058*	
C7	0.0592 (2)	1.28169 (10)	0.77585 (15)	0.0509 (4)	
H7	−0.0022	1.3178	0.7234	0.061*	
C12	−0.0253 (2)	0.67021 (10)	0.80237 (15)	0.0519 (5)	
H12	−0.0988	0.6519	0.7373	0.062*	
C11	0.0072 (2)	0.75257 (9)	0.81904 (14)	0.0456 (4)	
H11	−0.0452	0.7907	0.7658	0.055*	
H1O1	−0.266 (2)	0.9158 (11)	0.6408 (10)	0.068*	
H2O1	−0.291 (2)	0.9418 (13)	0.7474 (13)	0.068*	
H2O2	0.389 (3)	0.9826 (11)	1.0949 (17)	0.077 (7)*	
H1O2	0.544 (2)	1.021 (2)	1.134 (3)	0.149 (15)*	

### Atomic displacement parameters (Å^2^)

**Table:**

	*U*^11^	*U*^22^	*U*^33^	*U*^12^	*U*^13^	*U*^23^
O1W	0.0546 (9)	0.0784 (9)	0.0607 (9)	−0.0029 (7)	−0.0121 (7)	−0.0218 (7)
O1	0.0764 (10)	0.0408 (7)	0.0760 (9)	−0.0070 (6)	−0.0009 (8)	0.0042 (6)
O2W	0.0648 (10)	0.0722 (9)	0.0623 (9)	−0.0155 (8)	0.0000 (8)	−0.0202 (7)
N1	0.0494 (8)	0.0396 (7)	0.0464 (8)	−0.0076 (6)	−0.0043 (7)	−0.0017 (6)
N2	0.0468 (8)	0.0403 (7)	0.0440 (8)	−0.0064 (6)	−0.0088 (6)	−0.0030 (6)
N3	0.0636 (10)	0.0439 (8)	0.0487 (8)	−0.0001 (7)	−0.0054 (7)	0.0017 (6)
O2	0.0731 (9)	0.0519 (7)	0.0702 (9)	−0.0125 (6)	−0.0370 (8)	0.0024 (6)
C1	0.0695 (14)	0.0482 (10)	0.0957 (16)	−0.0127 (9)	0.0025 (12)	−0.0117 (10)
C2	0.0523 (10)	0.0401 (9)	0.0497 (9)	−0.0011 (7)	0.0111 (8)	−0.0009 (7)
C3	0.0489 (10)	0.0472 (9)	0.0450 (9)	−0.0052 (7)	−0.0023 (8)	−0.0042 (7)
C4	0.0500 (10)	0.0418 (9)	0.0439 (9)	0.0017 (7)	−0.0030 (8)	0.0007 (7)
C5	0.0427 (9)	0.0420 (8)	0.0386 (8)	−0.0005 (7)	0.0022 (7)	−0.0033 (6)
C8	0.0447 (9)	0.0457 (9)	0.0420 (9)	−0.0029 (7)	−0.0049 (7)	−0.0037 (7)
C9	0.0428 (9)	0.0436 (9)	0.0383 (8)	−0.0055 (7)	−0.0026 (7)	−0.0010 (6)
C10	0.0380 (8)	0.0421 (8)	0.0343 (8)	−0.0016 (7)	0.0009 (7)	−0.0026 (6)
C14	0.0518 (10)	0.0479 (9)	0.0375 (9)	−0.0021 (8)	−0.0068 (8)	−0.0020 (7)
C13	0.0578 (11)	0.0413 (9)	0.0546 (10)	−0.0017 (8)	−0.0021 (9)	−0.0064 (7)
C6	0.0478 (10)	0.0500 (10)	0.0441 (9)	0.0022 (8)	−0.0062 (8)	−0.0028 (7)
C7	0.0554 (11)	0.0477 (9)	0.0480 (9)	0.0074 (8)	−0.0003 (8)	0.0046 (7)
C12	0.0555 (11)	0.0484 (9)	0.0478 (9)	−0.0034 (8)	−0.0100 (8)	−0.0096 (8)
C11	0.0486 (10)	0.0442 (9)	0.0412 (9)	0.0013 (7)	−0.0068 (8)	0.0003 (7)

### Geometric parameters (Å, º)

**Table:**

O1W—H1O1	0.814 (9)	C3—H3	0.9300
O1W—H2O1	0.828 (9)	C4—C5	1.388 (2)
O1—C2	1.3672 (19)	C4—H4	0.9300
O1—C1	1.413 (2)	C5—C6	1.400 (2)
O2W—H2O2	0.830 (15)	C5—C8	1.450 (2)
O2W—H1O2	0.828 (17)	C8—H8	0.9300
N1—C8	1.270 (2)	C9—C10	1.493 (2)
N1—N2	1.3890 (17)	C10—C11	1.382 (2)
N2—C9	1.3451 (19)	C10—C14	1.381 (2)
N2—H2N2	0.8600	C14—H14	0.9300
N3—C14	1.335 (2)	C13—C12	1.370 (2)
N3—C13	1.335 (2)	C13—H13	0.9300
O2—C9	1.2199 (18)	C6—C7	1.368 (2)
C1—H1A	0.9600	C6—H6	0.9300
C1—H1B	0.9600	C7—H7	0.9300
C1—H1C	0.9600	C12—C11	1.379 (2)
C2—C7	1.383 (2)	C12—H12	0.9300
C2—C3	1.388 (2)	C11—H11	0.9300
C3—C4	1.382 (2)		
			
H1O1—O1W—H2O1	108.4 (17)	N1—C8—H8	119.0
C2—O1—C1	118.54 (14)	C5—C8—H8	119.0
H2O2—O2W—H1O2	111 (2)	O2—C9—N2	122.46 (14)
C8—N1—N2	115.97 (13)	O2—C9—C10	120.50 (14)
C9—N2—N1	117.85 (12)	N2—C9—C10	117.04 (13)
C9—N2—H2N2	121.1	C11—C10—C14	117.41 (14)
N1—N2—H2N2	121.1	C11—C10—C9	125.79 (14)
C14—N3—C13	116.33 (13)	C14—C10—C9	116.70 (13)
O1—C1—H1A	109.5	N3—C14—C10	124.64 (14)
O1—C1—H1B	109.5	N3—C14—H14	117.7
H1A—C1—H1B	109.5	C10—C14—H14	117.7
O1—C1—H1C	109.5	N3—C13—C12	123.60 (15)
H1A—C1—H1C	109.5	N3—C13—H13	118.2
H1B—C1—H1C	109.5	C12—C13—H13	118.2
O1—C2—C7	115.38 (14)	C7—C6—C5	121.87 (14)
O1—C2—C3	124.65 (14)	C7—C6—H6	119.1
C7—C2—C3	119.97 (14)	C5—C6—H6	119.1
C4—C3—C2	119.85 (14)	C6—C7—C2	119.58 (15)
C4—C3—H3	120.1	C6—C7—H7	120.2
C2—C3—H3	120.1	C2—C7—H7	120.2
C3—C4—C5	121.08 (14)	C13—C12—C11	118.95 (14)
C3—C4—H4	119.5	C13—C12—H12	120.5
C5—C4—H4	119.5	C11—C12—H12	120.5
C4—C5—C6	117.64 (14)	C12—C11—C10	119.05 (14)
C4—C5—C8	123.31 (14)	C12—C11—H11	120.5
C6—C5—C8	119.04 (13)	C10—C11—H11	120.5
N1—C8—C5	122.07 (14)		

### Hydrogen-bond geometry (Å, º)

**Table:**

*D*—H···*A*	*D*—H	H···*A*	*D*···*A*	*D*—H···*A*
N2—H2*N*2···O1*W*	0.86	2.08	2.9013 (17)	161
O1*W*—H1*O*1···N3^i^	0.81 (1)	2.08 (1)	2.8697 (18)	166 (2)
O1*W*—H2*O*1···O2*W*^ii^	0.83 (1)	1.93 (1)	2.749 (2)	171 (2)
O2*W*—H1*O*2···N1^iii^	0.83 (2)	2.40 (2)	3.209 (2)	166 (3)
O2*W*—H2*O*2···O2	0.83 (2)	2.01 (2)	2.8182 (17)	164 (2)

Symmetry codes: (i) *x*−1/2, −*y*+3/2, *z*−1/2; (ii) −*x*, −*y*+2, −*z*+2; (iii) −*x*+1, −*y*+2, −*z*+2.
